# Technical evaluation of the InBios Strongy Detect IgG ELISA assay for the diagnosis of *Strongyloides stercoralis* infection

**DOI:** 10.1186/s13071-024-06501-4

**Published:** 2024-12-23

**Authors:** Sara Roose, Marco Prato, Adama Kazienga, Iris Peelaers, Justien Arens, Gemechu Tadessa Leta, Cristina Mazzi, Dora Buonfrate, Bruno Levecke, Francesca Tamarozzi

**Affiliations:** 1 Ghent University, Merelbeke, Belgium; 2 IRCCS Sacro Cuore Don Calabria Hospital, Negrar di Valpolicella, Verona Italy; 3 Erasmus MC, University Medical Center Rotterdam, Rotterdam, The Netherlands; 4 Ethiopian Public Health Institute, Addis Ababa, Ethiopia

**Keywords:** *Strongyloides stercoralis*, Strongyloidiasis, Diagnosis, Serology, Seroassay, ELISA, Technical evaluation

## Abstract

**Background:**

Strongyloidiasis is a neglected tropical disease (NTD) caused by the soil-transmitted helminth *Strongyloides stercoralis,* recently included in the 2030 targets of the World Health Organization for the control of STHs. Assessment of infection prevalence is fundamental for decision-making about the implementation of control programs, but diagnostic assays to be applied in such context require evaluation. Seroassays based on recombinant antigens, which could be produced in a standardized and scalable manner, are particularly appealing for use in control programs. In this study, we performed a technical evaluation of the InBios Strongy Detect IgG ELISA, based on recombinant antigens NIE and SsIR, which has shown promising for field use.

**Methods:**

A total of 46 plasma samples from Ethiopian children were used for this technical evaluation. Repeatability was evaluated on duplicate samples per plate, on four plates per day for 3 days using Bland–Altman plots, analysis of residuals, and variance components analysis. Three samples were selected for evaluation of the uniformity of test results within a single plate (border effect) by two-sided *t*-test. Correlation between samples and internal ELISA positive controls was analyzed using Spearman’s rank correlation coefficient applied on the results of 777 samples analyzed with the assay in a previous field-based study.

**Results:**

Within and between plate residuals ranged from −0.05 to + 0.05 and −0.1 to + 0.1, respectively. Total variance was estimated at 0.327; 99.6% of variation could be attributed to the samples. There was no systematic border effect and a negligible correlation between positive internal control and samples results (*R*^2^ = 0.213; *p* < 0.001).

**Conclusion:**

The results obtained in this study, in highly controlled conditions, point toward the InBios Strongy Detect IgG ELISA assay being reproducible, with no systematic border effect. These results encourage further assay’s development and evaluation for use in practice, including determination of preset cutoff values for positivity, which is currently not provided.

**Graphical Abstract:**

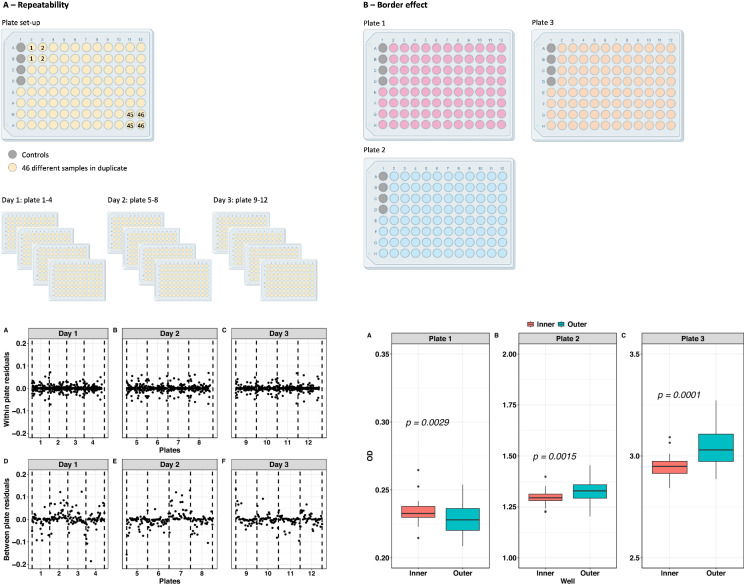

**Supplementary Information:**

The online version contains supplementary material available at 10.1186/s13071-024-06501-4.

## Background

Strongyloidiasis is a neglected tropical disease (NTD) caused by the soil-transmitted helminth (STH) *Strongyloides stercoralis*. It is estimated that 300–600 million people are infected, mostly in tropical and subtropical areas, especially impacting communities with inadequate access to water, sanitation, and hygiene [1]. The parasite is transmitted through skin penetration of infective larvae contaminating soil in areas where open defecation is practiced, and causes chronic infection through a peculiar auto-infective cycle. When clinically evident, strongyloidiasis manifestations range from intermittent or persistent gastrointestinal, dermatological, and respiratory symptoms to rapidly fatal hyper-infection in immunocompromised individuals, especially as the result of human T-lymphotropic virus infection or corticosteroid treatment [2].

Strongyloidiasis has been recently included in the 2030 targets of the World Health Organization (WHO) for the control of STHs [3]. The publication of guidelines for the implementation of control activities for strongyloidiasis and of the target product profile for new diagnostic assays to be used in such control programs is expected soon. Assessment of infection prevalence is fundamental for decision-making about the implementation and monitoring of these control programs. However, the status of infection with *S. stercoralis* can be difficult to ascertain, as detailed in a recent comprehensive review [4], and currently no gold-standard technique and no consensus exist on the diagnostic method of choice for the diagnosis of *S. stercoralis* infection for either epidemiological or clinical context. *Strongyloides*-specific methods are required for the diagnosis of this infection, since techniques commonly used for the diagnosis of other STHs (e.g., Kato–Katz) are inadequate for *S. stercoralis* [4].

In addition to intrinsic assay’s characteristics (e.g., sensitivity, specificity, and reproducibility), features such as acceptability and feasibility (e.g., sample management, ease of use, cost, and scalability) should be considered when deciding on a diagnostic test to be used in population-based activities. Stool-based assays (Baermann method, agar plate culture, and molecular assays) have low sensitivity due to the intermittent and variable larval output in stool; although highly specific, these assays are also labor intensive and also expensive in the case molecular assays [4]. Serology has generally higher sensitivity, although performances vary between assays and cross-reactions impact specificity [4]. The possibility to perform seroassays on samples obtained by finger prick, which could be used to test across multiple NTDs amendable for control [5], and the use of recombinant antigens, which could be produced in a standardized and scalable manner, make seroassays appealing for use in control programs.

Recently, the InBios Strongy Detect IgG ELISA (InBios International Inc., Seattle, WA, USA), based on the two *S. sterocoralis* recombinant antigens NIE and SsIR, was made available as research use only (RUO). In clinical-based diagnostic accuracy studies, the sensitivity ranged from 78% to 98.6%, while the specificity equaled 98%; the assay seemed also appropriate for post-treatment monitoring [6–8]. In a field-based diagnostic study, sensitivity and specificity were 83.5% and 91.7%, respectively, using sera obtained from dried blood spots [9]. The different study designs, populations [including background immune status, e.g., human immunodeficiency virus/human T-lymphotropic virus type 1 (HIV/HTLV) infection prevalence], sample collection strategies, and statistical analysis methods applied in these studies might be at the basis of these varied results, as well as other technical aspects such as the absence of a predefined cutoff value for positivity, which implies that the cutoff for assay results interpretation must be calculated at every use. In any case, this assay appears promising for use in population-based prevalence studies and control programmes.

In addition to accuracy results, technical knowledge about the characteristics of the assay is required for meaningful assay evaluation and data interpretation; therefore, this study aimed at evaluating the technical characteristics of the InBios Strongy Detect IgG ELISA.

## Methods

### Samples

For the technical evaluation, samples derived from those obtained from Ethiopian children enrolled in the study by Roose et al. (2022) were used [10]. These samples were collected in 2019, shipped to Belgium under controlled cold temperature, stored at −20°C thereafter, and had been thawed twice before thawing for use in this study. A total of 46 plasma samples showing a diverse range of optical density (OD) values using the same InBios Strongy Detect assay, and for which an adequate volume was available, were randomly selected among those analyzed in the context of a previous study aiming to map the prevalence of *S. stercoralis* in Ethiopia (unpublished data). Control sera from Belgian blood donors were used in the latter study to calculate the cutoff for positivity.

### ELISA test

The InBios Strongy Detect IgG ELISA is an enzymatically amplified sandwich immunoassay that detects IgG antibodies against *Strongyloides* recombinant antigens NIE and SsIR in serum. The test is currently available as RUO and requires storage at 4 °C. The assay was performed according to the manufacturer’s instructions. Positive and negative control samples are provided in the kit, together with quality control requirements, but no predefined cutoff for results interpretation is indicated. Quality control requirements were mean negative control OD < 0.300 at 450 nm reading, mean positive control OD > 0.500, and discrimination capacity (mean positive control OD/mean negative control OD) > 5. Briefly, samples and controls were diluted 1:100 in the kit’s sample buffer and 100 µL added to antigens-coated 96-well plate test wells. After 30 min incubation at 37 °C and wash, 100 µL horseradish peroxidase-conjugated anti-human-IgG secondary antibody was added, followed by a second incubation of 30 min at 37 °C. After washing, 100 µL of liquid TMB substrate was added. The reaction was stopped after 10 min by adding 50 µL of stop solution, and read by spectrophotometer (Infinite F50, Tecan) at 450 nm. No background (blank) adjustment was applied, following manufacturer’s instructions. Measurements exceeding the upper detection limit (overflow) were assigned the maximal OD value of 4.

### Repeatability

To evaluate the repeatability of the test results, 46 samples were tested in duplicate on four ELISA plates each day for 3 days (Additional file 1: Fig. S1A). Samples were diluted 1:10 using the sample dilution buffer in a single dilution plate before 1:100 dilution was applied into the ELISA plates using a multichannel pipette. This procedure minimized the likelihood of introducing variation from dilution procedures and the time difference between sample transfer onto the ELISA plates.

### Border effect

To evaluate the uniformity of test results within a single plate, three plates were used, each of them probed with an identical plasma sample in all its wells (except for the wells used for the internal controls), resulting in 92 replicates of the same sample (Additional file 1: Fig. S1B). The three samples were chosen to represent a range of OD values, with plate 1 testing a low OD sample (0.2), plate 2 a moderate OD sample (1.2), and plate 3 a high OD sample (3.0).

### Data analysis

The repeatability and the border effect were evaluated on the basis of the methods illustrated by Charlier et al. [11]. Between-duplicate repeatability was evaluated through Bland–Altman plot of duplicate measurements of each sample [12]. A total of 46 samples were tested in duplicate on 12 different plates, resulting in 552 data points. Within- and between-plate repeatability were explored by analysis of residuals. We further used variance components analysis to assess the repeatability over duplicates within plate, and between plates and days. To this end, the following model was used:

$${Y}_{ijkl}= \mu +{s}_{i}+{d}_{j}+{P}_{k\left(j\right)} +{e}_{ijkl}$$

here, ${Y}_{ijkl}$ represents the $lth$ measurement of sample $i$ on plate $k$ on day $j$ ($l=\text{1,2};i=1-46, k=1-4;j=1-3$), $\mu$ the overall mean, ${s}_{i}$ the effect of sample $i \sim$ $N\left(0, {\sigma }_{s}^{2}\right)$, ${d}_{j}$ the effect of day $j\sim N(0, { \sigma }_{d}^{2})$, ${P}_{k\left(j\right)}$ the effect of plate $k$ within day $j \sim N\left(0, {\sigma }_{p}^{2}\right)$, $\text{and} {e}_{ijkl}$ the random error term $\sim N\left(0, {\sigma }^{2}\right)$.

The application of different cutoff values for positivity, in terms of interpretation of the results of samples tested on different plates, was explored graphically by plotting median and interquartile ranges (IQR) of OD values per well across four plates per day. Two previously established cutoff values were used. The first cutoff value was defined as part of a previous study mapping the prevalence of *S. stercoralis* in Ethiopia (unpublished data) and was set at 0.26, calculated as the mean plus three standard deviations (SD) of the OD values obtained from 90 samples of a non-endemic population, specifically Belgian blood donors. The second cutoff value was 0.885, which is based on Tamarozzi et al. [6].

Border effect was evaluated by comparing the mean OD values of the outer wells (two wells from the border) with the mean OD of the inner wells, per each plate, by a two-sided *t*-test.

In a previous study carried out in Ecuador (the ESTRELLA study) evaluating the accuracy of the assay for field use in population studies [9], a noticeable variability in positive internal control OD values could be noted, although quality control requirements indicated by the manufacturer were always complied with. The correlation between sample results and internal positive control samples OD was analyzed using Spearman’s rank correlation coefficient applied on the results of 777 samples analyzed with the InBios assay in the ESTRELLA study. All analyses and graphical representations of the results were done using R version 4.3.2, R Studio (Posit, Boston, MA, USA) and Biorender.com.

## Results

In all cases, quality control requirements of the ELISA assay were met; six samples showed an OD result above the superior limit of reading (overflow) in at least one of the results of the repeatability experiment and were assigned the maximal OD value of 4 for the analysis. OD results closely matched those obtained using the same InBios Strongy Detect assay to map the prevalence of *S. stercoralis* in Ethiopia (unpublished data).

### Repeatability

**Fig. 1:**
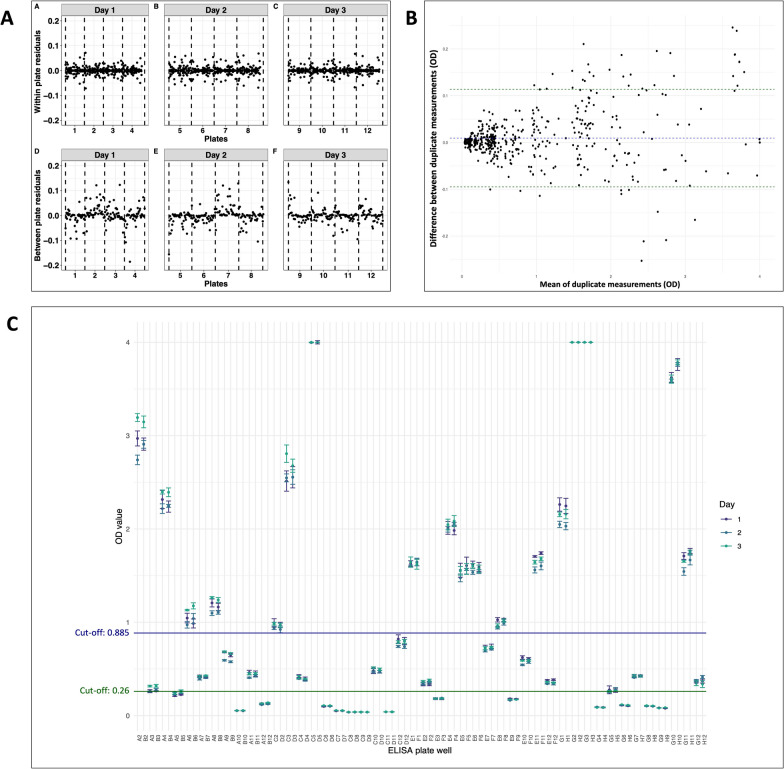
Repeatability evaluation. **A** Within- and between-plate residuals per plate and per day. **B** Bland–Altman plot of duplicate measurements of 46 samples on 12 different plates. The dashed blue line indicates the mean difference between the duplicate measurements. The dashed green lines provide the range in which 95% of the difference between duplicate measurements are expected to lie, assuming a normal distribution. **C** Median and interquartile ranges of OD values measured per well. Each pair of duplicate samples is positioned adjacently (e.g., well A2 and B2 held identical samples). The blue line indicates the cutoff value from the study by Tamarozzi et al. [6] (OD: 0.885). The green line cutoff was calculated as mean +3 × SD using serum samples from a non-endemic population (OD 0.26)

Within and between plate residuals per plate and per day are plotted in Fig. 1A. Both within- and between-plate residuals were approximately the same for all tested plates, ranging from −0.05 to + 0.05 (within-plate residuals) and −0.1 to + 0.1 (between-plate residuals). Total variance was estimated at 0.327; 99.6% of variation could be attributed to the samples (Table 1). Figure 1B shows the Bland–Altman plot of sample duplicates. Results were largely congruent, although a more evident dispersion could be observed with increasing OD values.

**Table 1:** Test repeatability over duplicates within plate, and between plates and days using random effect model

Source of variation	Variance	Percentage of total variance (%)
Sample	0.3262	99.60
Duplicate within a plate	0.0130	0.39
Plate within the same day	< 0.001	0.012
Day	< 0.001	< 0.01

The table illustrates the variance and the attributable variance expressed as the variance of each source of variation over the total variance

In the absence of a univocal cutoff for positivity for this assay, the relevance of between plate and day variability on sample result interpretation was evaluated by using two cutoff values previously obtained with two different methods and samples cohorts one from Ecuador [9], and one including Belgian donors and Ethiopian children (unpublished) (Fig. 1C). As expected, considering the results of the variance components analysis, only in a few cases the variability in OD for identical samples, observed across different plates or separate days, led to interpretations that alternated between positive and negative results. This was noted in just two samples when applying a cutoff of 0.885, and in three samples with the cutoff of 0.26.

### Border effect

**Fig. 2:**
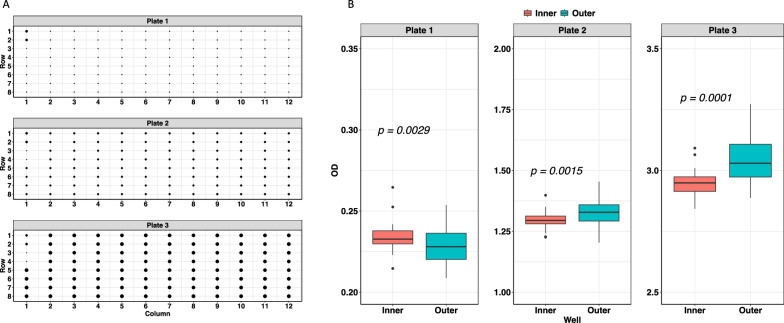
Border effect evaluation. **A** Set-up of the border effect evaluation. Each of the three ELISA plates contained one identical plasma sample in all their wells. ELISA kit’s internal positive controls are added in wells 1:1 and 2:1 and internal negative controls in wells 3:1 and 4:1 in each plate. The diameters of the dots represent the OD value. **B** Assessment of the border effect comparing the mean OD value between inner and outer wells across the three plates using a two-sided *t*-test

The OD values obtained from the three samples tested each on one of the three plates are plotted as dots diameters in Fig. 2A. There was no systematic difference in mean OD values between inner and outer wells: in two plates (2 and 3) inner wells had lower mean values than outer wells, while the opposite was observed for plate 1 (Fig. 2B).

### Correlation between internal positive control and samples results

The correlation between positive internal control OD and samples OD results was negligible (*R*^2^ = 0.213; *p* < 0.001; Additional file 2: Fig. 2) when evaluated on data obtained from 777 serum samples from a field study analyzed with the ELISA assay [9], which showed variable positive internal control OD values between plates.

## Discussion

Seroassays based on recombinant antigens have the advantage over crude or purified antigen-based tests of being potentially more reproducible, scalable, and specific, since they do not rely on biological material obtained from clinical or animal samples. In the case of strongyloidiasis, the difficulty in obtaining *S. stercoralis* larvae ex vivo and the current lack of a self-standing in vitro culture system make such recombinant antigen-based assays particularly appealing. Moreover, for strongyloidiasis, the use of seroassays for screening purposes is extremely convenient due to the drawbacks of stool-based assays required for its diagnosis, being poorly sensitive, labor intensive (microscopy-based techniques), and expensive (molecular assays) [4]. 

The InBios Strongy Detect IgG ELISA, based on recombinant antigens NIE and SsIR, has been evaluated for sensitivity and specificity in several previous studies [6, 8, 9], and showed promising for field use [9]; however, a rigorous technical evaluation was required, which could add information on its possible application.

In the present study, the assay proved reproducible, with no systematic border effect; the virtual totality of the within- and between-plate variability could be attributed to the samples themselves. Variability in OD results for the same sample was more evident with increasing OD values, which highlights the need for a careful identification of a cutoff for positivity to minimize different classification of the same sample, especially in mid-range OD values. The absence of guidance on cutoff value(s) from the manufacturer is a clear limitation of this assay, since it is not always possible to derive cutoff values at each test use.

In the present study, no predominant overflow OD result was observed, as opposed to what was obtained in a recent study evaluating the assay’s application for strongyoidiasis follow-up after treatment, which was performed with the same assay’s lot as the present study [7]. Whether this was due to the characteristics of the patient cohort from which the samples were derived in the study by Prato and colleagues [7] (i.e., all patients with confirmed *S. stercoralis* infection by microscopy or polymerase chain reaction, PCR) is difficult to know. None of the samples evaluated with the ELISA assay in the ESTRELLA study [9] resulted in an overflow result, including those with infection confirmed by microscopy or PCR; therefore, the conditions causing predominant overflow results in the study by Prato et al. [7] remain to be ascertained.

Variation in OD values obtained for the internal positive control has been observed in a previous study [9], which posed the question as to whether the same factors at the basis of this result would influence the OD values of analyzed samples. Since no such issue was observed in the present study, we analyzed the results obtained in the above-mentioned previous study [9] and found no relevant correlation between the positive internal control OD and samples’ OD results. Therefore, variability of positive controls OD values seems to be due to intrinsic characteristics of the control itself, and not related to the assay’s plate. Since the kit’s provided positive and negative controls are not used to calculate the cutoff value for positivity, but only used for the assay’s quality check, the internal controls’ variability also does not appear at the basis of the different cutoff values that were calculated in different studies [6, 9], including this one. Therefore, while our results point toward the assay being reproducible, further studies are needed to define cutoff values for positivity to be used in practice. In principle, this should be done with the use of area-specific controls, because of the heterogeneity in immune responses apparently observed across different populations, which may impact on the diagnostic accuracy of the assay . However, while the use of area-specific controls could be envisaged to this aim, it would impact on the easiness and wideness of the assay’s employability.

This study has several limitations. First, as mentioned above, the infection status of the Ethiopian children from whom the samples were obtained was unknown. Therefore, we could not evaluate sensitivity, specificity, and cross-reactivity of this assy using this samples cohort. Second, the same ELISA assay’s lot was used; therefore, we could not evaluate the between-lot reproducibility of results. Third, the experiments were conducted by very experienced technicians and extreme care was taken to minimize any possible source of variation not related to the assay itself (e.g., dilution procedures, samples plating, incubation procedures, etc.). Therefore, the robustness of such results should be confirmed in a similar study performed in less controlled conditions. Nevertheless, as mentioned above, our results point toward the assay being reproducible and within- and between-plate variability being attributable to the samples themselves, and encourage further studies to define cutoff values for positivity to be used in practice. Finally, although the manufacturer indicates that the ELISA assay should be used with serum, the use of plasma, available for this study, evidently appears to not having impacted the technical performance of the assay. However, a systematic parallel comparison of performances using plasma and serum should be performed.

## Conclusions

The results obtained in this study point toward the InBios Strongy Detect IgG ELISA assay as being reproducible, with no systematic border effect. These results encourage further assay’s development and evaluation for use in practice, including determination of preset cutoff values for positivity. Development of a rapid test format using the same antigens to further meet population-based activities applicability would also be encouraged.

## Supplementary information

Additional file 1: Figure S1. Experimental plate set-up. Panel A: repeatability experiment. Panel B: border effect experiment. Control samples were included in all plates for quality control.

Additional file 2: Figure S2. Correlation between positive internal control OD and samples OD. *X*-axis: OD value of the positive internal control. *Y*-axis: OD results of samples obtained in the ESTRELLA study in Ecuador [9]. Correlation coefficient equalled 0.213, demonstrating negligible correlation.

## Data Availability

The dataset generated during the current study is available in Zenodo repository at https://zenodo.org/records/11211752.

## Abbreviations

IQR: Interquartile range
NTD: Neglected tropical disease
OD: Optical density
RUO: Research use only
STH: Soil-transmitted helminth
WHO: World Health Organization
