# Supplementary material for: Technical evaluation of the InBios Strongy Detect IgG ELISA assay for the diagnosis of Strongyloides stercoralis infection
Source: Parasit Vectors. 2024 Dec 23;17:534. doi: 10.1186/s13071-024-06501-4 (PMC11667801; doi:10.1186/s13071-024-06501-4)
Supplement: Supplementary file 1 — Additional file 1: Figure S1. Experimental plate set-up. Panel A: repeatability experiment. Panel B: border effect experiment. Control samples were included in all plates for quality control. [file 13071_2024_6501_MOESM1_ESM.pdf]

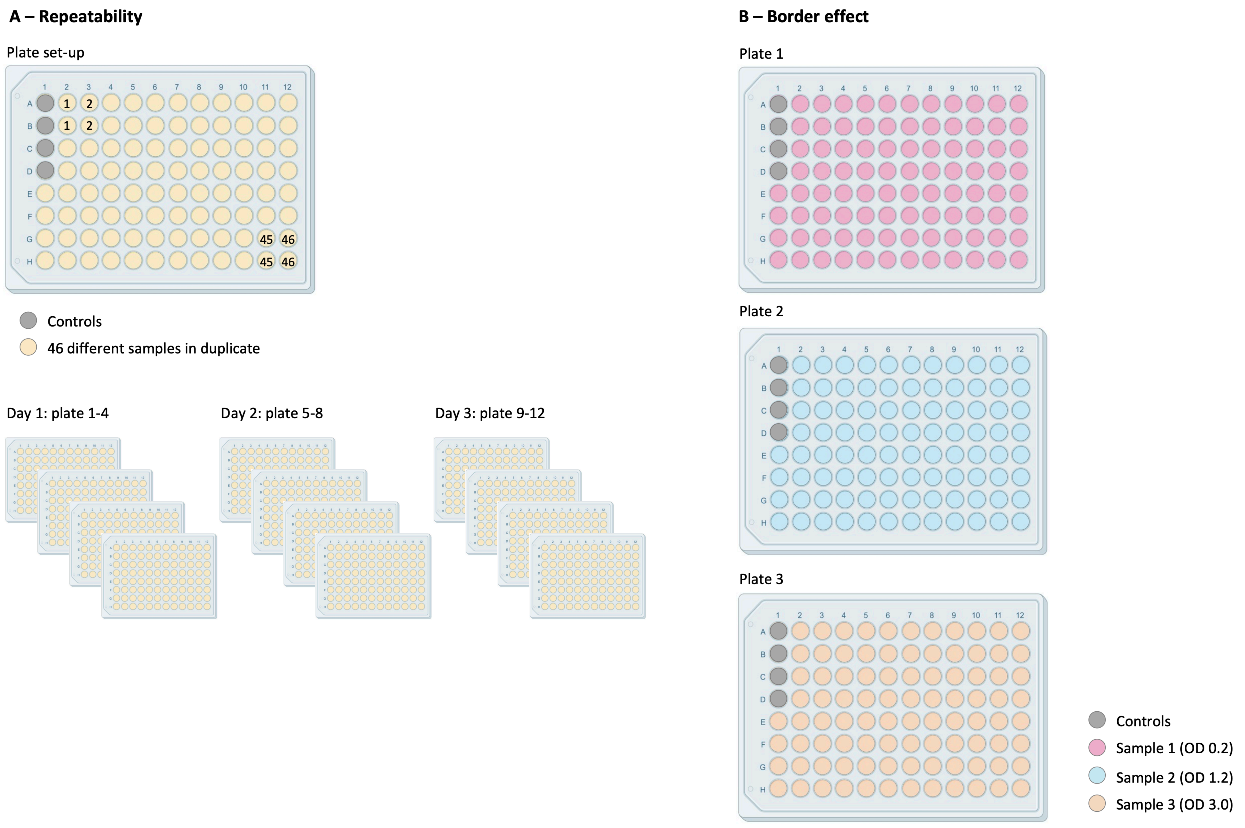
A – Repeatability
Plate set-up
Controls
46 different samples in duplicate
Day 1: plate 1-4
Day 2: plate 5-8
Day 3: plate 9-12
B – Border effect
Plate 1
Plate 2
Plate 3
Controls
Sample 1 (OD 0.2)
Sample 2 (OD 1.2)
Sample 3 (OD 3.0)
