# Supplementary figures and images for: Technical evaluation of the InBios Strongy Detect IgG ELISA assay for the diagnosis of Strongyloides stercoralis infection
Source: Parasit Vectors. 2024 Dec 23;17:534. doi: 10.1186/s13071-024-06501-4 (PMC11667801; doi:10.1186/s13071-024-06501-4)

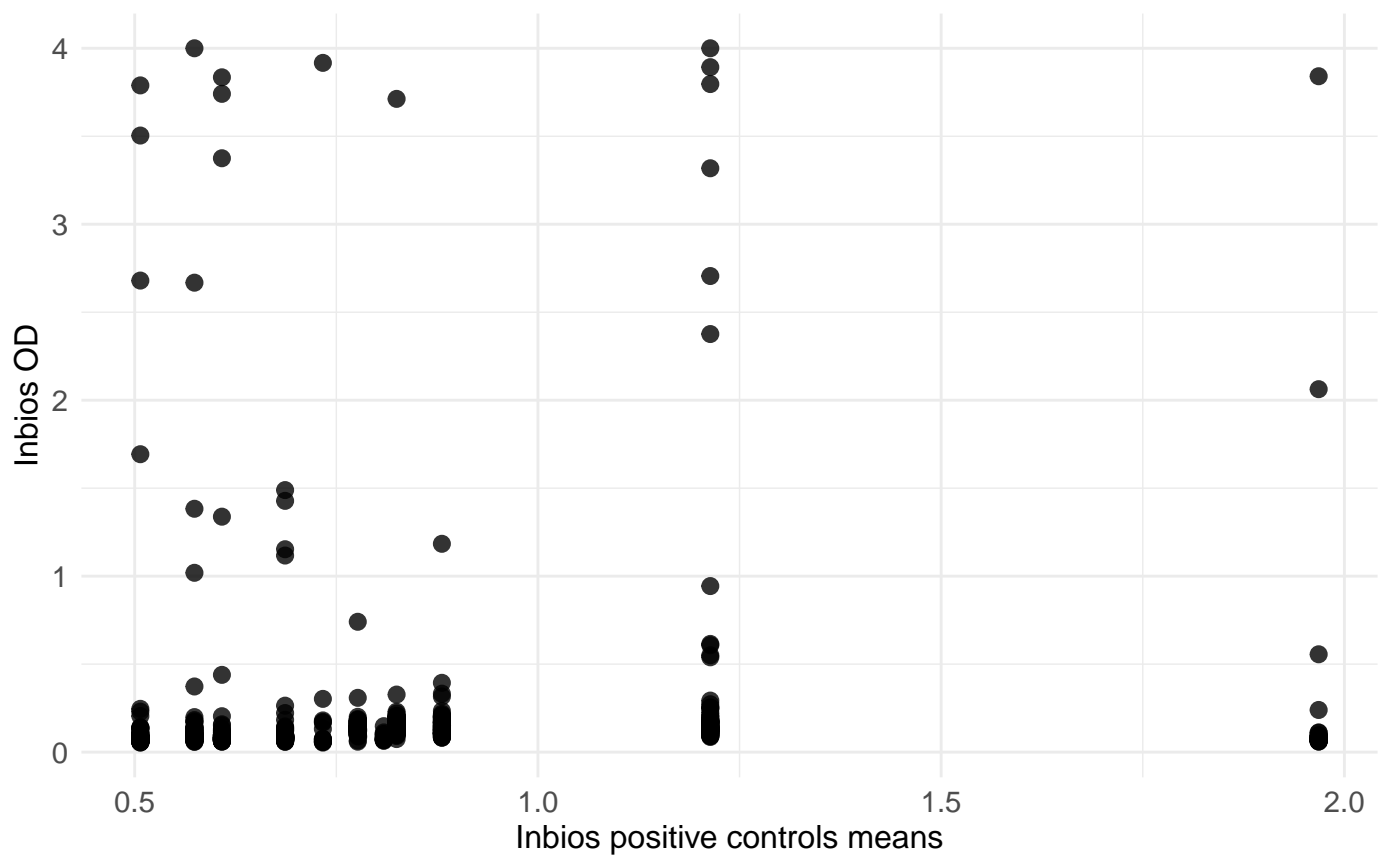

Supplement: Supplementary file 2 — Additional file 2: Figure S2. Correlation between positive internal control OD and samples OD. X-axis: OD value of the positive internal control. Y-axis: OD results of samples obtained in the ESTRELLA study in Ecuador [9]. Correlation coefficient equalled 0.213, demonstrating negligible correlation. [file 13071_2024_6501_MOESM2_ESM.pdf]
